# The study of a total and two hypothalamic-specific BBS10 knockout models highlights the importance of systemic inactivation in the obese phenotype in Bardet Biedl Syndrome

**DOI:** 10.1186/2046-2530-4-S1-P5

**Published:** 2015-07-13

**Authors:** M Scerbo, F Costa, C Obringer, H Dollfus, V Marion

**Affiliations:** 1 Laboratoire de Génétique Médicale, INSERM, UMR-U1112, Université de Strasbourg, Strasbourg, France; 2 Hôpitaux Universitaires de Strasbourg, Strasbourg, France

## 

The autosomal recessive disorder, Bardet-Biedl syndrome (BBS) is an iconic ciliopathy, clinically characterized by obesity, retinopathy, polydactyly and renal dysfunction. To date, 19 *BBS *genes have been identified, with BBS10 being one of the most commonly mutated genes in human patients. The historical origin of the BBS-induced obesity has been associated with leptin resistance correlated with hyperleptinemia as well as decreased signaling in the appetite-governing arcuate nucleus neurons (ARC) of the hypothalamus. The ARC controls energy homeostasis, food intake and energy expenditure, through the detection of peripheral hormones by POMC and AgRP/NPY expressing neurons. POMC neurons are anorexigenic while NPY/AgRP are orexigenic, and both are ciliated cells. Several hormone receptors have ciliary localization, as the leptin receptor, and inactivation of the BBS proteins results in their mislocalization and signaling impairment. The present work aims at investigating the origins of obesity in the BBS by comparing the phenotype of a *BBS10 *total knockout (*Bbs10^-/-^*) with that of two *BBS10 *hypothalamic-specific KO mice: namely the POMC (*Bbs10^fl/fl^;POMC-Cre^+/-^*) and the AgRP (*Bbs10^fl/fl^;AgRP-Cre^+/-^*). *Bbs10^-/- ^*mice develop obesity, together with other BBS cardinal traits, but surprisingly, both *Bbs10^fl/fl^;AgRP-Cre^+/- ^*and *Bbs10^fl/fl^;POMC-Cre^+/- ^*display a lean phenotype. Further characterizations of these mice highlighted the activation of compensatory mechanisms in response to the specific BBS10 inactivation probably forestalling the obese phenotype. These results indicate the complexity of the BBS-related obese phenotype, and support the need for an integrative approach that would include the contribution of other peripheral organs to better understand the origins of obesity in BBS.

